# *Cyamopsis tetragonoloba* Gum‐Based Active Coatings Incorporated With *Pycnocycla bashagardiana* Essential Oil for Reducing Postharvest Losses of Fresh *Pistachio* Fruits

**DOI:** 10.1002/fsn3.70388

**Published:** 2025-07-07

**Authors:** Mohammed Hamdan Aldarraji, Hatem Mohammed Hasan, Hossein Talepour Ardekani, Mojtaba Heydari‐Majd, Heidar Meftahizade, Mansour Ghorbanpour

**Affiliations:** ^1^ Plant Protection College of Agriculture, University of Misan Amarah Iraq; ^2^ Department of Medicinal and Industrial Plants College of Medicinal and Industrial Plants, Kirkuk University Kirkuk Iraq; ^3^ Graduate in Financial Management, Islamic Azad University, Science and Research Branch Tehran Iran; ^4^ Faculty of Agriculture & Natural Resources, Ardakan University Ardakan Iran; ^5^ Department of Horticultural Sciences Faculty of Agriculture & Natural Resources, Ardakan University Ardakan Iran; ^6^ Department of Medicinal Plants Faculty of Agriculture and Natural Resources, Arak University Arak Iran

**Keywords:** edible coating, guar gum, pistachio nuts, *Pycnocycla bashagardiana* essential oil, quality characteristics

## Abstract

Fresh pistachios, prized for their distinct flavor and aroma, suffer from limited postharvest shelf life due to fungal contamination, particularly aflatoxin‐producing species. This study investigates the efficacy of guar coatings (0.25%, 0.5%, and 1%), incorporating *Pycnocycla bashagardiana* essential oil (PBEO) with varying concentrations (50, 100, and 200 μL), in extending the storage life of fresh pistachios over 60 days. The physicochemical properties of the coating solutions (zeta potential (ζ), particle size (PD) and contact angle (CA)) were characterized, alongside pistachio quality attributes including weight loss, peroxide value (PV), phenolic content (PC), titratable acidity (TA), texture firmness, antioxidant capacity, and total soluble solids (TSS). Antifungal activity against *Aspergillus* and *Penicillium* was also assessed. GC/MS and GC‐FID analysis identified E‐beta‐(ocimene), Myristicin, Z‐beta (‐ocimene), and oleic acid as major components and fatty acids profile of PBEO and guar gum, respectively. The FTIR spectrum of guar gum reveals the presence of key functional groups within the polysaccharide structure. Increasing the concentration of PBEO in the solution preparation led to a significant increase in PSD, ζ, and CA magnitude. Results indicate that PBEO‐loaded guar coatings significantly enhanced PC and antioxidant capacity, reduced weight loss and PV, maintained TSS and firmness, and retarded loss of TA. 200 μL PBEO‐loaded guar at 0.25% effectively inhibited *Penicillium* growth, while 50 μL demonstrated optimal control of both *Aspergillus* and *Penicillium*. The PBEO‐loaded guar coating extended pistachio shelf life from 30 to 60 days based on microbial counts.

## Graphical abstract

Treated pistachio retained phenolics and total antioxidant capacity. The guar gum‐PBEO coating had the best antifungal effects on Penicillium and Aspergillus growth. The guar gum, along with PBEO, elevates the storage period of fresh pistachio up to 60 days.

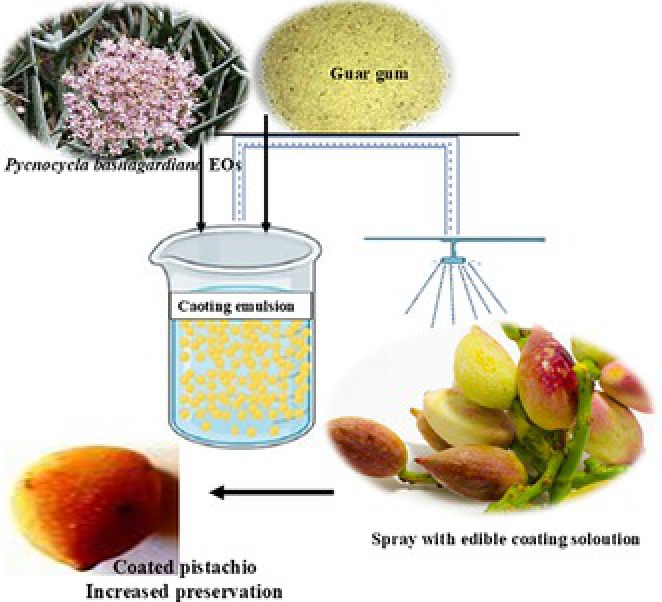

## 1 Introduction

The global pistachio market represents a substantial agricultural sector, with an estimated annual economic value of approximately US$2 billion and a total production volume approaching one million metric tons (Özdemir and Aksoy 2024). Between 1994 and 2020, worldwide pistachio production experienced a marked increase, rising from 345,408 tons to 1.1 million tons, with the United States, Turkey, and Iran collectively accounting for approximately 70% of this output (Nejatian et al. 2023). Pistachios are not only economically important, contributing significantly to the economies of producing nations, but also highly valued for their nutritional composition (Sharefiabadi et al. 2025). This nut is characterized by a high concentration of essential amino acids and monounsaturated fatty acids, exceeding that of other commonly consumed nuts, which may contribute to beneficial effects on cholesterol levels. Furthermore, pistachios are a source of protein, dietary fiber, vitamins, minerals, and various bioactive compounds, including carotenoids and phenolic compounds (Shakerardekani and Saberi 2024; Özdemir and Aksoy 2024). Fresh pistachios are particularly favored by consumers during the harvest season due to their distinctive sensory characteristics, including a unique flavor profile and appealing aroma. The desirable soft texture, delicate taste, and pleasant fragrance further enhance their nutritional value and overall consumer appeal (Jalali et al. 2025). Raw pistachios are commercially available in both hulled and unhulled forms, with the former generally being more economically accessible and widely traded during the harvest period. However, despite their desirable organoleptic properties, the elevated moisture content of fresh pistachios renders them susceptible to microbial proliferation, thereby limiting their shelf life. Moreover, the potential for contamination by aflatoxin‐producing molds, notably *Aspergillus flavus* and *Penicillium* species, represents a significant food safety concern (Pravallika et al. 2025). Therefore, the application of natural preservation and processing techniques is paramount to mitigate spoilage and maintain product quality.

In this context, plant‐derived compounds, particularly essential oils (EOs), have emerged as promising natural antimicrobial agents (Bahrami et al. 2022). These EOs, extracted from diverse plant sources, contain a complex array of secondary metabolites exhibiting bactericidal, virucidal, fungicidal, and antioxidant activities, potentially rivaling those of synthetic pharmaceutical compounds (Bahrami et al. 2023; Bautista‐Hernández et al. 2025). EO composition in *Pycnocycla bashagardiana*, an Iranian endemic within the Apiaceae family, varies significantly depending on the plant's developmental stage (Ghazazani et al. 2023). The highest essential oil concentration (2.22%) is reported during flower initiation, characterized by a chemical profile dominated by p‐cymene, E‐beta‐ocimene, myristicin, sabinene, (Z)‐beta‐ocimene, and isomyristicin (Kaur et al. 2024). Despite limited existing applications in fruit storage, the inherent antimicrobial properties of *P. bashagardiana*, coupled with its high myristicin content, warrant further investigation into its potential as a natural food preservative. While direct incorporation of EOs into food products offers a simple approach to preservation, it is often constrained by the potential for chemical decomposition of the EOs and concomitant alterations in the product's organoleptic profile, necessitating the exploration of alternative delivery strategies (Heydari‐Majd et al. 2019a, 2019b).

Innovations in delivery systems, such as natural biopolymers, enhance the efficacy of these natural preservatives by providing sustained release and additional protective properties against oxidation and microbial spoilage (Heydari‐Majd et al. 2023; Sharefiabadi et al. 2025). Delivery systems, such as edible films and coatings, can improve antimicrobial efficacy by maintaining elevated surface concentrations of active compounds and controlling their diffusion rates (Shakerardekani et al. 2021). Numerous studies have documented the application of EOs from *Zataria multiflora* EO and pistachio green hull EOs in coating formulations aimed at extending the shelf life of fruits and vegetables (Hashemi et al. 2021a, 2021b; Nejatian et al. 2023). Direct application methods, including spraying or dipping, can be effective, particularly when combined with the application of edible coatings. Furthermore, composite systems incorporating various biopolymers and active compounds offer a comprehensive approach to product protection. These technologies have demonstrated success in extending the shelf life and maintaining the quality of fresh fruits and vegetables (Bremenkamp and Sousa Gallagher 2025).

Within the diverse array of biopolymers available, *Cyamopsis tetragonoloba* L. gum (guar gum) stands out as a particularly promising material for applications in food and fruit packaging. The structural properties of guar gum, specifically its high molecular weight polysaccharides with a mannose:galactose ratio of 2:1, provide a robust matrix suitable for a range of packaging and coating applications (Rani et al. 2025). Furthermore, the presence of various phytochemicals, including alkaloids, carbohydrates, phenols, proteins, and tannins, contributes to its potential antioxidant and antimicrobial activities, which are critical for extending the shelf life of perishable food products (Rani et al. 2025). Research has shown that guar gum‐based edible coatings can effectively enhance the postharvest quality and shelf life of fruits, as demonstrated in studies with tomatoes (Ruelas‐Chacon et al. 2017) and hazelnut sauce (Pourfarzad and Taleb Derakhshan 2021). The ability of guar gum to form hydrogels and films offers opportunities for controlled release of preservatives or antioxidants, potentially leading to improved food storage conditions (Dallabrida et al. 2024). Although traditional applications of guar gum have primarily centered on its therapeutic and industrial uses, its potential in the realm of food preservation and packaging is receiving increasing attention.

Recognizing the absence of existing research on the antioxidant and antimicrobial potential of *P. bashagardiana* essential oil (PBEO) as a natural additive in edible coatings for extending the shelf life of fresh produce, this study sought to develop a sustainable solution. Therefore, this study aimed to exploit the antifungal properties of PBEO and the encapsulating capabilities of guar gum to develop a more robust and safer hydrophobic coating, aligning with contemporary industrial demands for environmentally friendly and sustainable materials. Given the limited storability of fresh pistachios, this research examined the impact of varying guar gum concentrations, in conjunction with three different concentrations of PBEO, on postharvest shelf life and key quality attributes of pistachios during a storage period of up to 60 days.

## 2 Materials and Methods

### 2.1 Collection of Plant Materials

**FIGURE 1:**
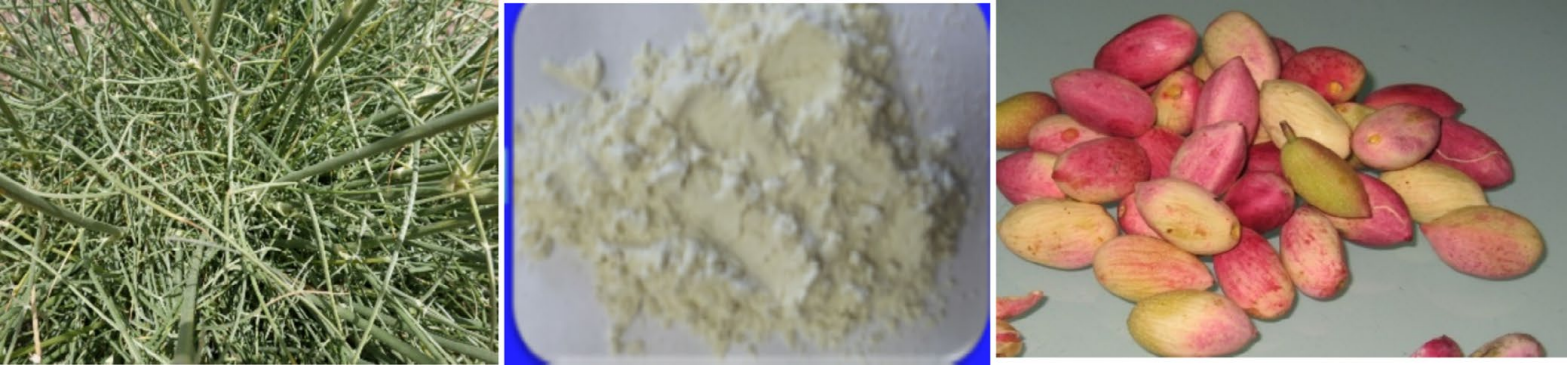
*Pycnocycla bashagardiana*, *Cyamopsis tetragonoloba* L., and *Pistachio vera*.

This experiment was conducted in 2024 at the laboratory of the faculty of agriculture, Ardakan University, located at latitude 32.31° N and longitude 54.01° E. The essential oil of *Pycnocycla bashagardiana* was collected from Bashagard village, Jask County, Hormozgan Province, Iran (25°38′38″ N, 57°46′28″ E, 900 m). The guar gum was prepared from the medicinal plant research center, Ardakan University, and *pistachio* samples (fruit cv. “Ahmad‐Aghaei”) were obtained from the botanical herbal orchards in Yazd. Prior to collection, plants were visually inspected to ensure they were healthy and free from infection. The fruits were washed under running tap water to remove dust and other particulate matter, thoroughly cleansed, and subsequently dried (Figure 1). Healthy, fresh pistachios were separated from unripe or damaged ones. Approximately 500 g of fresh pistachios was used for each sample.

### 2.2 Essential Oil Extraction

After cleaning, the samples were dried in the shade at room temperature and then powdered (50 g of each part were used for essential oil extraction). EOs extraction was performed using the hydrodistillation method with a Clevenger apparatus (Naui and Khaldi 2025). The resulting essential oil was dehydrated by separating it from the water surface using anhydrous sodium sulfate. After weighing and calculating the essential oil production yield, it was stored in sealed glass containers in a refrigerator.

#### 2.2.1 GC/MS Analysis

To identify and separate the compounds present in the essential oil, GC/MS devices were used. A Varian 3400 gas chromatograph connected to a mass spectrometer with an ion trap detector and an ion trap system and ionization energy in the mass spectrometer of 70 eV, with a 5% DB semi‐polar column, 30 m long, 0.25 mm internal diameter, 0.25 μm stationary phase layer thickness, column head gas pressure of 35 psi, temperature of 40°C–250°C with an increase rate of 4°C per minute, injection chamber temperature of 260°C, and transfer line temperature of 270°C were used. The identification of the compounds present in the essential oil was performed using retention indices and examining the proposed mass spectra (Ben Chira et al. 2025).

### 2.3 Extraction of Gum Content and Fatty Acid Determination

To extract gum content, a separation process was performed, adapting the methodology of Khurizadeh et al. (2024). Seeds were initially hydrated by soaking in water for 8 h. Following this, seed shells were removed through manual rubbing. Subsequently, mechanical pressure was applied to separate the gum from the embryo and endosperm. The separated gum was then dried in an oven at 50°C for 8 h.

The identification of fatty acids was carried out using gas chromatography, according to the method of Chiofalo et al. (2018). In this method, fatty acid methyl esters (FAMEs) were prepared from lipids using a methanol:sulfuric acid mixture (9:1 v/v) in screw‐capped tubes. After esterification, the mixture was cooled, and hexane was added to extract the FAMEs. The hexane layer, containing the FAMEs, was then transferred to chromatography vials for analysis. FAME analysis was performed using gas chromatography with flame ionization detection (GC‐FID) on an Agilent 6890N system. Separation was achieved using a 30 m × 0.25 mm i.d. Omegawax capillary column (Supelco) with a 0.25 μm film thickness. The temperature program began at 160°C (6 min hold), increased to 250°C at a rate of 3°C/min, and held at 250°C for 10 min. Injector and detector temperatures were maintained at 250°C. One microliter of sample was injected with a split ratio of 1:50, using helium as the carrier gas at a flow rate of 1 mL/min. Fatty acid identification was based on comparing retention times to known FAME standards (Supelco). Peak areas were quantified using ChemStation software (Agilent). Fatty acid concentrations were expressed as a percentage of total identified FAMEs. Each sample was analyzed in triplicate. The Atherogenic Index (AI) and Thrombogenic Index (TI) were calculated from the identified fatty acid profiles.

#### 2.3.1 FTIR Spectroscopy of Guar Gum

Fourier transform infrared (FTIR) spectroscopy was employed to investigate the functional groups present in guar gum, with spectra recorded on a Perkin Elmer FTIR spectrometer (USA) over the wavenumber range of 400–4000 cm^−1^ at a resolution of 4 cm^−1^ (Niazmand et al. 2025).

### 2.4 Preparation of Guar Gum‐Essential Oil‐Based Edible Coating

To prepare the guar gum–EO‐based edible coating, we followed a modified version of the method outlined by Rezaiyan Attar et al. (2023). Guar gum solutions, at concentrations of 0%, 0.25%, 0.5%, 0.75%, and 1% (w/v), were formulated by dissolving guar gum in a water solution and PBEO solutions, at concentrations of 0, 50, 100, and 200 μL. A control solution, without guar gum essential oils, was also prepared.

### 2.5 Physical Properties of PBBE‐Guar Gum Coating Solution

The zeta potential (ζ) of the samples was determined after dilution in deionized water (1:100 v/v) using a ZetaSizer Nano ZS (Nanotrac Wave II, USA) equipped with folded capillary cells (DTS1070). The measurements were performed over a zeta potential range of −200 to +200 mV. Additionally, the particle size (PS) was analyzed using a Mastersizer 3000 (Nanotrac Wave II, USA) equipped with a Hydro dispersion unit, with measurements conducted at a temperature of 25°C.

### 2.6 Wettability of BPEO‐Guar Gum Coatings on Fruits

The wettability contact angle (θ) was determined using a Dynamic Contact Angle measuring device, employing the sessile drop method. In this method, a droplet of the deionized water was deposited onto a horizontal surface via a needle with an internal diameter of 1.20 mm. All measurements were completed within 10 s. To ensure reliability, at least 12 replicate measurements were conducted for each parameter and formulation (Sapper et al. 2019).

### 2.7 Coating the Fresh Pistachio Fruit

Each coating was applied to the pistachio kernel using the method described by Hashemi et al. 2021a, 2021b. The pistachios were sprayed with each coating solution using a manual sprayer and then stored in an oven at 25°C for 1 h in order to dry. Each treatment unit consisted of 150–200 g of pistachio kernel (approximately 50–60 fruits), which were placed in sealed polypropylene containers (10 × 15 × 10 cm) after treatment and drying. A control treatment, devoid of coating, was also prepared to assess the effects of edible coating on storage life. The treated pistachios were then stored at 2°C±1°C and 85% ± 5% relative humidity (RH) for up to 60 days, with quality evaluations conducted at 10‐day intervals during cold storage.

### 2.8 Investigated Parameters

The evaluated parameters of *Pistacia vera* included acidity, kernel and shell firmness, weight loss, phenolic content, peroxide value, microbial contamination, and antioxidant capacity.

#### 2.8.1 Measurement of Weight Loss

Percentage weight loss in pistachio samples was determined by comparing the initial weight to the weight at each storage time point, using the formula: Weight loss (%) = ((Initial weight − Weight at storage time)/Initial weight) × 100 (Afrashteh et al. 2023).

#### 2.8.2 Measurement of Peroxide Value

To evaluate oil quality, fresh pistachio kernels were initially dried at 40°C for 48 h. Subsequently, oil was extracted from the ground kernels utilizing the procedure outlined by Zhang et al. 2021. The peroxide value (PV) content of the extracted oil was then quantified according to the established AOCS methods Cd 8–53 and Cd 3d‐63 (Zhang et al. 2021).

#### 2.8.3 Measurement of Total Phenolic Content

Fresh pistachio kernels and hulls were individually frozen in liquid nitrogen and stored at −20°C prior to total phenolic content (TPC) analysis. TPC was determined using the method of Heydari‐Majd et al. 2019a, 2019b, with results expressed as grams of gallic acid equivalents per kilogram of fresh weight (g kg^−1^).

#### 2.8.4 Determination of Titratable Acidity

Titratable acidity, expressed as malic acid, was determined through titration with 0.1 N sodium hydroxide. A 2.5 mL aliquot of fruit extract was diluted with 47.5 mL of distilled water and subsequently titrated to a pH endpoint of 8.2 using sodium hydroxide, following the method of Hosseinial Hashemi et al. (2021).

#### 2.8.5 Kernel and Shell Firmness

Pistachio fruit tissue firmness was measured using a handheld penetrometer (model 0585SN, manufactured in the Netherlands) with a 1 cm diameter piston. Measurements were taken at the equatorial region of the fruit after the peel was removed, and the results were expressed in kilograms per square centimeter (kg/cm^2^) (Afrashteh et al. 2023).

#### 2.8.6 Antioxidant Capacity

The antioxidant activity of *pistacia vera* extract was determined using the free radical substrate 1,1‐diphenyl‐2‐picrylhydrazyl, DPPH (LOBA Chemie, India) according to the described method of Marvdashti et al. (2023). The absorbance of the reaction mixture was measured at 517 nm using a T60 UV–visible spectrophotometer (Leicestershire LE17 5BH, UK). Comparisons were prepared using 50 μM 80% methanol instead of the extract. The percentage of DPPH radical inhibition (antioxidant activity) by the extract was calculated based on the following equation (Equation (1)):

(1) $$\%\text{Inhibition}=\frac{A-b}{A}\times 100$$

where A = absorbance of control and B = absorbance of extract sample (Marvdashti et al. 2023).

#### 2.8.7 Total Soluble Solids (TSS)

The total soluble solids (TSS) content was determined as the average of 10 pistachios per replicate using a digital refractometer (ATAGO, PAL‐1 model, Japan) (Hashemi et al. 2018).

#### 2.8.8 Shell Color

Pistachio shell color was assessed using a Konica Minolta CR‐400 Chroma Meter (Japan), following the methodology outlined by Hashemi et al. 2021a, 2021b. For each replicate, the color coordinates L*, a*, and b* were recorded for 20 individual pistachios. The total color difference (Δ*E*) resulting from sample covering was calculated using the following Equation (2):

(2) $$∆E=\sqrt{{\left(L_{t}-L_{0}\right)}^{2}+{\left(a_{t}-a_{0}\right)}^{2}+{\left(b_{t}-b_{0}\right)}^{2}}$$

where *L*_0_, *a*_0_, and *b*_0_ represent the initial L*, a*, and b* values of the pistachio shell, and *L*_*t*_, *a*_*t*_, and *b*_*t*_ are the corresponding values measured at subsequent time points.

### 2.9 Microbial Contamination Analysis

Mold and yeast populations in fresh pistachios were quantified as log colony‐forming units per gram (log CFU g^−1^) following a method adapted from Sabaghi et al. (2015). Briefly, 10 g of pistachio samples was crushed and homogenized with 90 mL of sterile 8.5 g/L NaCl solution using a stomacher (Stomacher 400, Seward Laboratory Blender, UK) for 2 min. Serial ten‐fold dilutions were prepared in sterile saline solution. For microbial enumeration, 1 mL aliquots from each dilution were pour‐plated in triplicate onto yeast extract glucose chloramphenicol (YGC) agar (Merck, Germany). Plates were incubated at 25°C for 5 days, and mold and yeast colonies were subsequently counted. The detection limit of this method was below 1 log CFU/g.

### 2.10 Statistical Analysis

Analysis of variance of the data was performed with SAS 9.2 software. Also, the comparison of data averages was performed using Duncan's test at the 5% level. Excel software was used to record data and draw graphs.

## 3 Results

### 3.1 Identification of Compounds in the Essential Oil of *P. bashagardiana* Flowers

**FIGURE 2:**
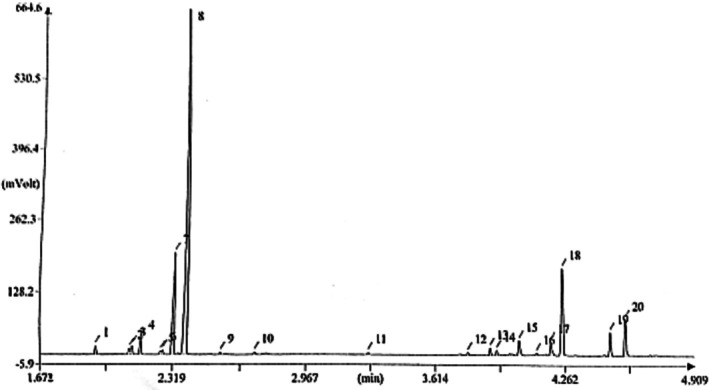
Chromatogram of the essential oil of the *Pycnocycla bashagardiana*.

About 19 compounds were found in PBEO (Figure 2). The main compounds were shown in Table 1. Analysis of the PBEO revealed a chemical profile dominated by E‐beta‐(ocimene) (54.3%), Myristicin (12.8%), and Z‐beta (−ocimene) (12.4%), cumulatively accounting for 79.5% of the total oil volume. This composition, comprising 14 identified compounds representing 96.86% of the oil, highlights a significant presence of monoterpene hydrocarbons and phenylpropanoids within the oil (Figure 2). These findings align with the broader understanding of the Apiaceae family's chemical diversity, as noted by Jahandar et al. (2018) and Yari et al. (1999), which emphasize the presence of diverse secondary metabolites, including EOs rich in various compound classes. Our identification of minor components like Humulene epoxide, Isomyristicin, and Linalool butyrate, though present in low concentrations, contributes to the comprehensive understanding of PBEO chemical diversity (Table 1). These compounds, while not dominant, may still contribute to the overall biological activity of the EO. The literature also highlights the importance of phenylpropanoids in PBEO, with myristicin being a primary constituent (Jahandar et al. 2018). Our study provides valuable insights into the EO composition of PBEO from Bashagard village, confirming the presence of key compounds like E‐beta‐ocimene and myristicin.

**TABLE 1:** Chemical components (%) of the essential oils distilled from *Pycnocycla bashagardiana*.

No.	Essential oil compounds	(%)
1	Beta‐pinene	0.96
2	Delta‐3‐carene	0.51
3	E‐beta‐(ocimene)	54.3
4	E‐caryophyllene	0.55
5	Humulene epoxide	4.96
6	Lavendulyl acetate	0.37
7	Isomyristicin	2.91
8	Lavendulyl isovalerate	1.91
9	Linalool butyrate	2.13
10	Methyl eugenol	0.33
11	Myrcene	1.91
12	Myristicin	12.8
13	Sabinene	0.81
14	Z‐beta (‐ocimene)	12.41

### 3.2 Fatty Acid Determination

**FIGURE 3:**
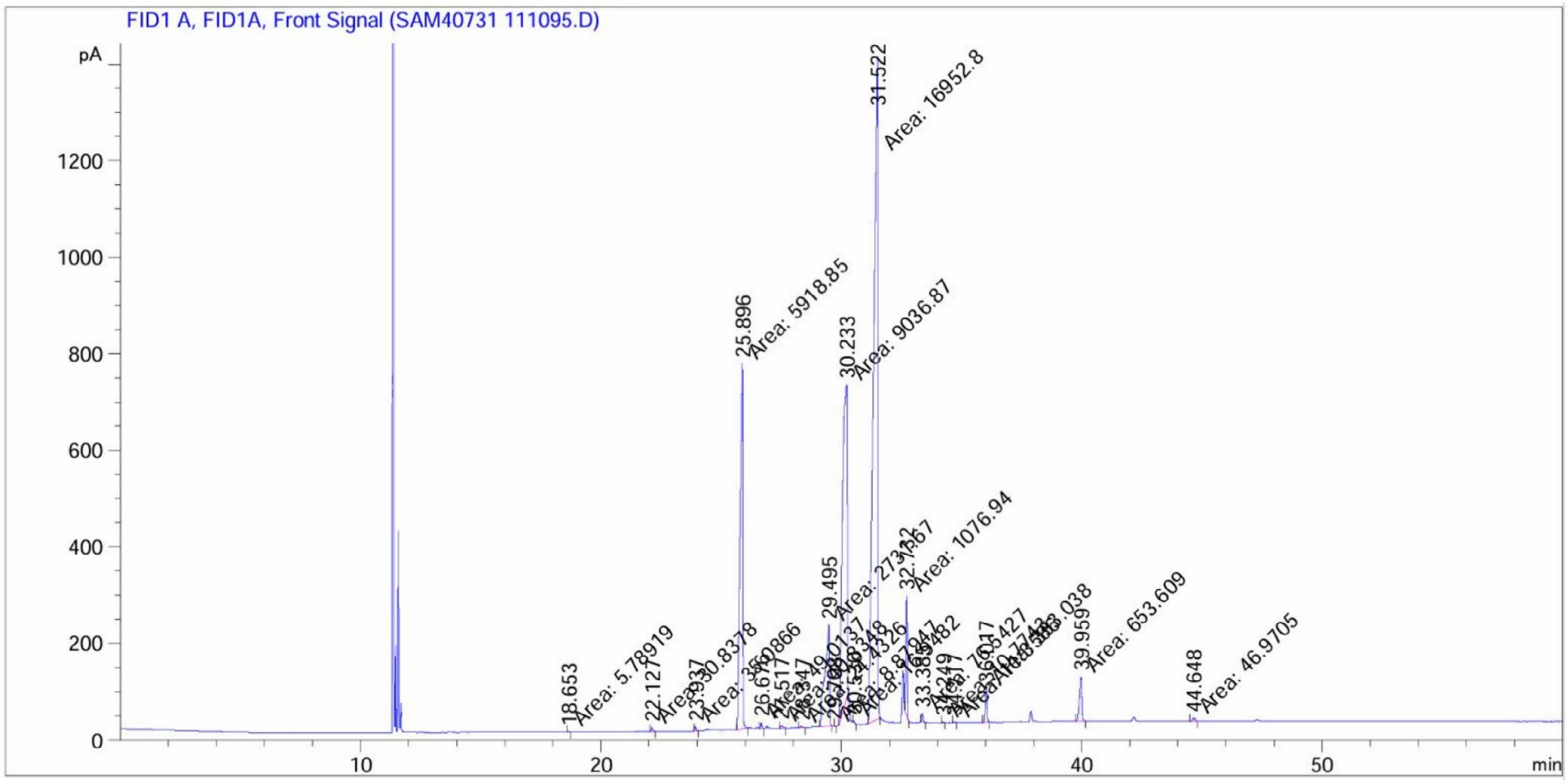
A flame ionization detection (GC‐FID) of Fatty acids in guar gum.

The profiles of fatty acid in guar gum showed in Chromatograph (Figure 3). Oleic acid devoted the maximum amount of fatty acids, as well as saturated fatty acids with 26.3% and unsaturated fatty acids with 73.58% were the composition of the fatty acid profile (Table 2).

**TABLE 2:** The results of fatty acids in guar gum.

Fatty acid	Amount (g/100 g)
Palmitic acid	15.96
Stearic acid	7.36
Lignoseric acid	1.96
Oleic acid	24.37
Linolenic acid	3.1
Behnic acid	0.95
Palmitoleic acid	0.25
Margaric acid	0.13
Louric acid	0.01
Miristic acid	0.08
Penta decanoic acid	0.09
Saturated fatty acid	26.3
Unsaturated fatty acid	73.58

### 3.3 FTIR Spectrum of Guar Gum

**FIGURE 4:**
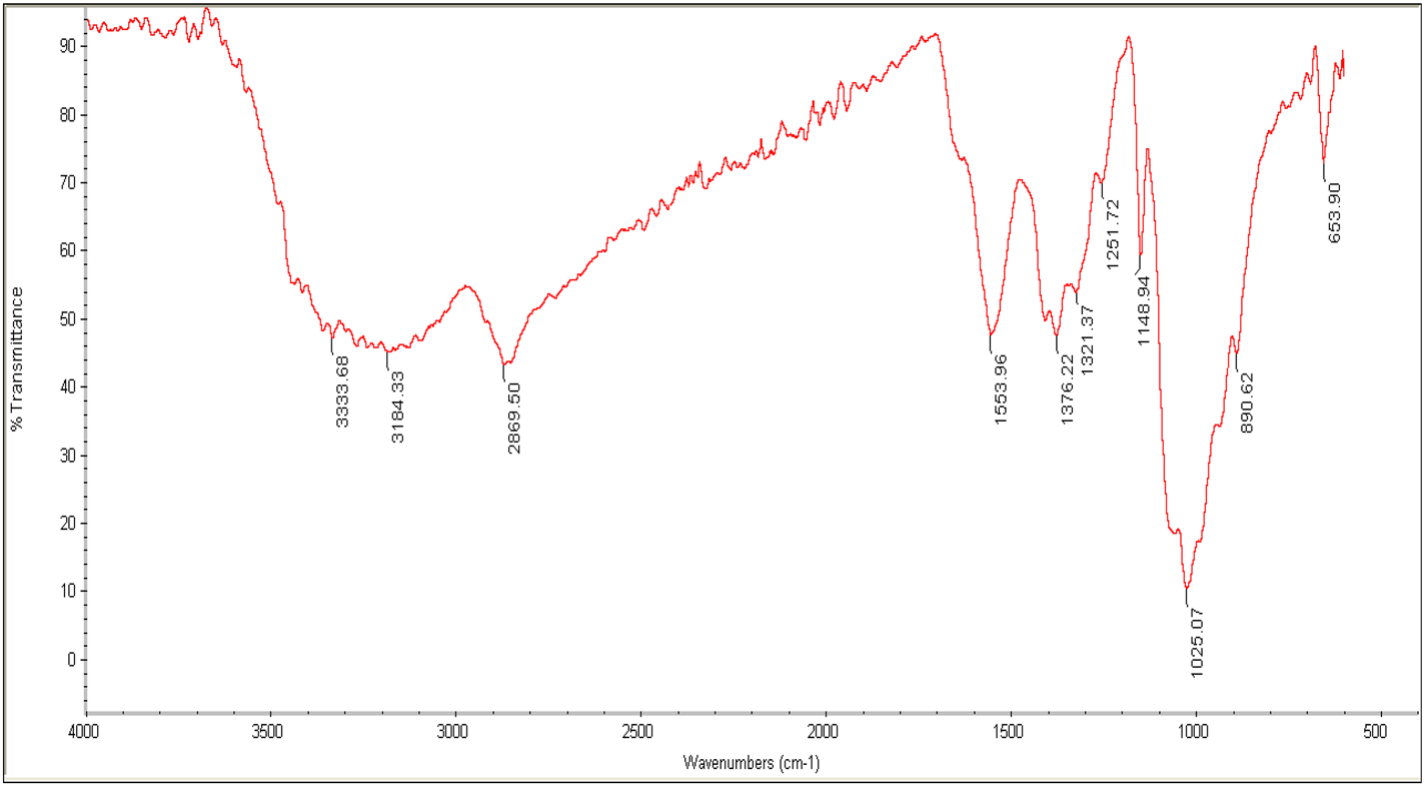
FTIR of guar gum.

The FTIR spectrum of guar gum reveals the presence of key functional groups within the polysaccharide structure, which play a crucial role in its physicochemical properties. The absorption bands in the range of 3200–3600 cm^−1^, with distinct peaks at 3333.68 cm^−1^ and 3184.33 cm^−1^, correspond to the stretching vibrations of O‐H bonds, indicating the abundance of hydroxyl groups and the presence of strong hydrogen bonding interactions within the galactomannan framework of this compound. In the 2800–3000 cm^−1^ region, the peak observed at 2869.50 cm^−1^ is attributed to the stretching vibrations of C‐H bonds in aliphatic polysaccharide chains, highlighting the organic and saturated nature of this biopolymer (Figure 4). In the 1500–1800 cm^−1^ region, the absorption band at 1553.96 cm^−1^ is likely associated with the stretching vibrations of carbonyl (C = O) groups, which may correspond to uronic acids or amide‐containing structures within guar gum. The presence of this band suggests possible structural modifications due to chemical alterations or environmental interactions. Additionally, in the 1300–1600 cm^−1^ range, the peaks at 1376.22 and 1321.37 cm^−1^ are indicative of C‐H bending vibrations and carboxylate functional groups, which are critical for understanding the chemical composition and structural properties of this polysaccharide (Figure 4).

In the fingerprint region (600–1500 cm^−1^), the prominent absorption peaks at 1251.72 cm^−1^, 1148.94 cm^−1^, and 1025.07 cm^−1^ confirm the presence of C–O stretching vibrations in glycosidic linkages, further supporting the galactomannan structure of guar gum. The absorption band at 890.62 cm^−1^ is likely associated with out‐of‐plane C‐H bending vibrations, commonly found in polysaccharide structures. Lastly, the peak at 653.90 cm^−1^ may correspond to specific bending vibrations of polymeric bonds, which contribute to the structural stability and complex nature of this compound (Figure 4).

### 3.4 Particle Size (PS) and Zeta Potential (ζ)

The properties of solutions, including PS and ζ, are critical determinants of coating performance. To elucidate the relationships between PBEO concentration and solution characteristics, the impact of varying levels of PBEO on the properties of coating solutions was investigated (Table 2). The PS and ζ of the pure guar gum solution were found to be 480 nm and −13.2 mV, respectively. These values are comparable to those reported by Pu et al. (2019), who obtained a ζ value of approximately −15 mV for pure guar gum. The results of this study revealed that the incorporation of PBEO significantly (*p* < 0.05) affected the PS and ζ of the coating solutions. Specifically, increasing the concentration of PBEO in the solution preparation led to a significant (*p* < 0.05) increase in both PS and ζ magnitude (Table 3). The PS range increased from 480.7 to 680.6 nm as the PBEO concentration increased, likely due to hydrophobic‐hydrophilic interactions between the PBEO and the guar gum matrix. The negative ζ values indicated a stable dispersion of the matrix components. Notably, the increasing concentration of PBEO improved the stability of the coating, as evidenced by the enhanced ζ values. The highest stability was achieved with the 200 μL PBEO formulation, which exhibited a ζ value of −16.7 mV, followed by the 100 μL PBEO (ζ = −15.6 mV) and 50 μL PBEO (ζ = −14.43 mV) formulations, respectively.

**TABLE 3:** Particle size distribution (PSD), zeta potential (ζ) and contact angle of pure guar gum solution and guar gum solutions containing the *Pycnocycla bashagardiana* essential oil (PBEO).

Sample	Particle size (nm)	Zeta potential (mV)	Contact angle (θ)
Guar gum	480.7 ± 1.20d	−13.2 ± 0.40d	60.10 ± 0.20d
Guar gum +50 μL PBEO	630.5 ± 0.92c	−14.4 ± 0.02c	75.20 ± 0.15c
Guar gum +100 μl PBEO	651.2 ± 0.90b	−15.6 ± 0.01b	87.60 ± 0.85b
Guar gum +200 μL PBEO	680.6 ± 0.85a	−16.7 ± 0.04a	90.15 ± 0.50a

*Note:* Different letters in the same column indicate significant differences (*p* < 0.05).

### 3.5 Contact Angle of BPEO‐Guar Gum Coatings on Fruits

When a droplet of water is deposited onto the smooth surface of a coating matrix, it forms an equilibrium contact angle (θ) with the surface. For hydrophilic surfaces, θ is typically less than 90°, whereas higher angles are indicative of less hydrophilic surfaces. The effect of PBEO on the CA value with water as the polar solvent is presented in Table 3. Notably, the pure guar gum coating exhibited a relatively low water CA value of 60.10°, indicating a high degree of hydrophilicity. However, the incorporation of PBEO led to a significant increase in CA, suggesting a reduction in surface hydrophilicity. These findings are consistent with previous reports on starch‐gellan coatings containing thyme EO (Sapper et al. 2019) and guar gum/chitosan edible films reinforced with orange EO (Cai et al. 2025), which also demonstrated changes in CA values in response to the addition of EOs.

### 3.6 The Effect of Time and Different Concentrations of PBEO on Physicochemical Parameters of *Pistacia vera*

Our study revealed that the weight loss of pistachios in the control group increased significantly from day one (48.8%) to the sixtieth day (26.7%). However, the application of PBEO at a concentration of 100 μL effectively mitigated this weight loss, reducing it to 30.6% (the end of storage) and consequently enhancing the storage quality of pistachios until 60 days (Table 3). Similarly, the PV, a marker of lipid oxidation, showed a substantial increase in the control group, rising from 0.214 (meq O_2_ kg^−1^ oil) on day one to 1.900 (meq O_2_ kg^−1^ oil) at the end of storage. Notably, the application of PBEO oil at 200 μL significantly reduced this peroxide value to 1.325 at the end of storage, further contributing to improved pistachio storage (Table 4). In contrast, the phenol content, indicative of antioxidant capacity, decreased in the control group from 7.18 g kg^−1^ on Day 1 to 0.46 g kg^−1^ on Day 60. Nevertheless, the application of PBEO at 200 μL effectively preserved the phenol content, preventing a drastic reduction and maintaining it at 1.25 g kg^−1^ at the end of storage, thereby enhancing storage stability (Table 4). Furthermore, the acidity of pistachios in the control group increased from 0.062% to 0.221% over the 60‐day period. However, the application of PBEO at 100 μL significantly limited this increase, resulting in an acidity of 0.171% and improving storage quality (Table 4). Regarding textural properties, the shell hardness of pistachios in the control group decreased from 7.23 to 3.73 kg N. Conversely, the application of PBEO at 100 μL effectively minimized this reduction, maintaining shell hardness at 3.24 kg N at the end of storage and contributing to better storage (Table 4). Finally, the kernel hardness of pistachios in the control group increased from 16.39 to 55.85 kg N. However, the application of PBEO at 100 μL significantly minimized this increase, maintaining kernel hardness at 60.23 Kg. N at the end of storage, which further enhanced the overall storage quality of pistachios (Table 4). In the control group, the antioxidant levels of pistachios exhibited a significant decrease from 84.65% on Day 1 to 8.87% at the end of storage. However, the application of 200 μL of PBEO effectively mitigated this decrease, maintaining antioxidant levels at 16.68%. This preservation of antioxidant capacity contributed to enhanced pistachio storage until 60th (Table 4).

**TABLE 4:** Evaluation of the effect of different concentrations of PBEO on the quality characteristics of fresh pistachios.

Time (days)	Essential oil (μL)	Weight loss (%)	PV (meq O_2_ kg^−1^ oil)	PC (g kg^−1^)	Acidity (%)	Shell firmness (kg N)	Kernel firmness (kg N)	Antioxidant capacity
1st	Control	48.8a	0.214m	7.18a	0.062j	7.23d	16.39jk	84.65b
	50	48.3a	0.234l	6.45b	0.021k	7.62cd	17.87j	85.98b
	100	49.5a	0.218l	6.37b	0.028k	7.85cd	16.80k	90.85ab
	200	48.5a	0.198n	6.98ab	0.026k	7.78cd	16.90k	93.26a
10th	Control	47.6a	0.447i	4.53d	0.059j	10.85a	18.15i	57.21d
	50	46.8b	0.298k	4.65d	0.058ij	10.65a	19.87i	55.23de
	100	47.2ab	0.315ij	3.89e	0.061i	10.23ab	20.48h	47.12e
	200	44.4c	0.369i	5.49c	0.057j	11.65a	21.65gh	63.54c
20th	Control	41.9e	0.798g	1.68ij	0.523a	7.08d	21.87gh	25.87j
	50	41.2e	0.702h	3.25ef	0.189f	8.23bc	23.49g	42.57g
	100	43.1d	0.766gh	3.42f	0.208de	9.65b	27.87ef	45.89f
	200	40.9f	0.725h	3.36f	0.210de	8.95bc	26.80f	43.54fg
30th	Control	37.1i	1.118d	1.85j	0.507a	5.65ef	26.85f	17.87k
	50	37.4i	0.798f	2.89gh	0.239b	5.87ef	26.11f	28.68i
	100	38.9g	0.815ef	2.15i	0.233c	6.10e	33.45de	25.87j
	200	37.9gh	0.809f	3.12g	0.238b	5.87e	34.24e	35.85h
40th	Control	31.9l	1.299c	0.89l	0.165gh	4.47f	35.15de	12.47m
	50	32.7k	1.125d	1.56k	0.164gh	4.23f	38.85d	17.88k
	100	35.6j	0.897ef	1.35kl	0.143h	4.19fg	48.98c	18.48k
	200	33/7k	0.912e	1.75j	0.140h	3.85fg	49.57c	23.68jk
60th	Control	26.7n	1.900a	0.46n	0.221d	3.73gh	55.85b	8.87n
	50	27.6mn	1.458ab	0.88m	0.175efg	2.87i	53.97c	14.87m
	100	30.6l	1.356b	0.93m	0.171gh	3.24h	60.23ab	13.68mn
	200	28.5m	1.325b	1.25kl	0.198fgh	2.75i	65.65a	16.68l

*Note:* The same letters in each column indicate no significant difference between groups.

Abbreviations: PC = Phenolic content, PV = peroxide value.

### 3.7 The Effect of Time and Different Concentrations of Guar Gum on Shelf‐Life Parameters in *Pistacia vera*

The results indicate that guar gum has a significant effect on preserving and improving the flavor index throughout the storage period. In the early phase (Day 15), the 0.25% guar concentration had the most positive impact on the flavor index. However, in the later stages (Days 40 and 60), increasing the guar gum concentration (0.5% and 1%) led to better flavor retention. In contrast, the control group without guar gum showed a significant decrease in the flavor index, highlighting the positive effect of this additive in improving the sensory characteristics of the product over time (Table 5). The results showed that the TSS content during the storage period (0, 15, 40, and 60 days) was affected by different concentrations of guar gum, showing different trends at each time point. On Day 0, the TSS value in the control group was very high (16.32), while in samples containing guar gum, the TSS levels were significantly lower (ranging from 2.55 to 2.95). On Day 15, the TSS value in the control group decreased compared to Day 0, reaching 5.62, while in guar gum‐treated samples, the TSS value varied between 3.70 and 4.08. On Day 40, a decreasing trend in TSS was observed in guar gum‐containing samples (ranging between 3.80 and 5.12). At the end of storage, the highest TSS value among the guar gum‐treated samples was recorded at 0.25% concentration, indicating that this concentration can help better preserve soluble compounds (Table 5).

**TABLE 5:** The effect of different concentrations of guar gum at various times on the quality characteristics of fresh pistachios.

Time (days)	Guar Gum (%)	Flavor Index	TSS (°Brix)	Acidity (%)	Weight loss (%)
0	Control	0.06l	16.32a	0.52n	0.86i
	0.25	0.07J	2.77n	0.56m	0.28p
	0.5	0.07h	2.95l	0.49o	0.30o
	1	0.07g	2.55o	0.59i	0. 72l
15th	Control	0.04p	5.62d	0.56m	0.77k
	0.25	2.01a	4.08i	0.61e	0.58n
	0.5	0.08d	3.70k	0.06h	0.65m
	1	0.05n	3.80j	0. 57l	1.03g
40th	Control	0.06m	6.65c	0.58k	1.08f
	0.25	0.07k	5.12f	0.60g	0.82j
	0.5	0.86c	4.40h	0.62d	0.91h
	1	0.08f	3.80j	0.67b	1.28d
60th	Control	0.04o	7.12b	0.58j	1.40b
	0.25	0.07i	5.55e	0.60f	1.32b
	0.5	0.08e	4.97g	0.62c	1.22e
	1	0.09b	2.87m	0.67a	1.74a

*Note:* Different letters in the same column indicate significant differences (*p* < 0.05).

The acidity level in the control group showed an increasing trend over time, reaching 0.58 by Day 60. The addition of guar gum led to an increase in acidity, with the highest acidity recorded in the 1% guar gum sample (0.67) at the end of storage. An unexpected acidity value (0.06) was observed in the 0.5% guar gum sample on Day 15. Overall, the use of guar gum, especially at higher concentrations, resulted in increased acidity, which may influence the sensory characteristics and shelf life of the product (Table 5).

Throughout the experiment, the highest weight loss was recorded in the control group, indicating the role of guar gum in reducing weight loss in the product. Samples containing 0.25% and 0.5% guar gum performed better in limiting weight loss compared to the control. However, increasing the guar gum concentration to 1% resulted in higher weight loss, especially in the later stages of the experiment (1.74 on Day 60). Ultimately, 0.5% guar gum appears to be the most effective concentration for controlling weight loss, while using 1% may lead to increased weight reduction (Table 5).

### 3.8 Shell Color

The rapid color change observed in fresh pistachio shells can negatively impact consumer acceptance, necessitating its evaluation across different treatment groups. Similar to other quality attributes, coating treatments, particularly those incorporating PBEO‐loaded guar, demonstrated the potential to stabilize shell color. Statistical analysis revealed that both surface treatments and storage duration significantly (*p* < 0.05) influenced the color indices (L*, a*, and Δ*E*) of fresh pistachios. Table 6 illustrates the changes in surface color, represented by L*, a*, and Δ*E*, observed in fresh pistachio shells during 60 days of storage. In general, L* and a* values decreased significantly (*p* < 0.05) across all treatments during storage.

**TABLE 6:** The effect of guar gum and PBEO at different times on pistachio shell color.

Time (days)	Guar gum (%)	Essential oil (μL)	L*	a*	Δ*E*
15	Control	Control	50 ± 0.20ab	27 ± 0.10a	4 ± 0.20i
		50	51 ± 0.10a	26 ± 0.20ab	3 ± 0.30ig
		100	52 ± 0.40a	27 ± 0.20a	4 ± 0.07i
		200	49 ± 0.20b	28 ± 0.06a	5 ± 0.20h
	0.25	Control	40 ± 0.10cd	23 ± 0.10c	6 ± 0.10g
		50	40 ± 0.09cd	22 ± 0.07c	6 ± 0.03g
		100	43 ± 0.20c	23 ± 0.20c	7 ± 0.10fg
		200	41 ± 0.40cd	24 ± 0.01b	7 ± 0.20fg
	0.5	Control	44 ± 0.09c	23 ± 0.06c	6 ± 0.10g
		50	45 ± 0.20c	24 ± 0.20c	7 ± 0.10fg
		100	47 ± 0.20b	24 ± 0.10c	7 ± 0.20fg
		200	45 ± 0.10c	26 ± 0.30ab	8 ± 0.30f
	1	Control	45 ± 0.40c	25 ± 0.08b	7 ± 0.08fg
		50	47 ± 0.20b	25 ± 0.10b	6 ± 0.10g
		100	48 ± 0.10ab	26 ± 0.30ab	8 ± 0.20f
		200	47 ± 0.20b	27 ± 0.10a	8 ± 0.30f
40	Control	Control	34 ± 0.20ef	19 ± 0.20d	9 ± 0.10de
		50	34 ± 0.40ef	18 ± 0.08de	9 ± 0.40de
		100	36 ± 0.20e	18 ± 0.20de	8 ± 0.10f
		200	38 ± 0.30d	20 ± 0.40d	9 ± 0.30de
	0.25	Control	36 ± 0.20e	18 ± 0.30de	10 ± 0.07d
		50	38 ± 0.30d	18 ± 0.20de	11 ± 0.20c
		100	39 ± 0.20d	17 ± 0.09e	10 ± 0.30d
		200	38 ± 0.40d	20 ± 0.10d	12 ± 0.40c
	0.5	Control	36 ± 0.09e	20 ± 0.30d	9 ± 0.20de
		50	37 ± 0.20de	19 ± 0.10d	9 ± 0.40de
		100	39 ± 0.40d	22 ± 0.10c	10 ± 0.20d
		200	38 ± 0.20d	21 ± 0.40d	10 ± 0.30d
	1	Control	38 ± 0.40d	20 ± 0.09d	10 ± 0.10d
		50	38 ± 0.10d	20 ± 0.30d	9 ± 0.10de
		100	39 ± 0.10d	21 ± 0.06d	11 ± 0.10c
		200	39 ± 0.30d	20 ± 0.30d	11 ± 0.40c
60	Control	Control	25 ± 0.30i	14 ± 0.09 g	15 ± 0.20a
		50	25 ± 0.20i	15 ± 0.20f	14 ± 0.09a
		100	28 ± 0.40h	14 ± 0.40 g	14 ± 0.10a
		200	29 ± 0.10g	15 ± 0.20f	13 ± 0.20b
	0.25	Control	28 ± 0.20g	15 ± 0.30f	11 ± 0.20dc
		50	28 ± 0.10g	16 ± 0.06f	10 ± 0.08d
		100	29 ± 0.40g	16 ± 0.20f	12 ± 0.10c
		200	31 ± 0.20f	17 ± 0.40e	12 ± 0.20c
	0.5	Control	28 ± 0.20g	15 ± 0.40f	11 ± 0.07d
		50	29 ± 0.20fg	15 ± 0.30f	11 ± 0.20d
		100	31 ± 0.10f	17 ± 0.20e	12 ± 0.20c
		200	31 ± 0.10f	18 ± 0.20de	13 ± 0.04b
	1	Control	29 ± 0.30fg	16 ± 0.20ef	12 ± 0.40c
		50	29 ± 0.20fg	15 ± 0.40f	11 ± 0.20d
		100	31 ± 0.40f	18 ± 0.09de	12 ± 0.10c
		200	30 ± 0.10f	18 ± 0.20de	13 ± 0.20b

*Note:* Different letters in the same column indicate significant differences (*p* < 0.05).

The decline in L* values indicates a darkening of the pistachio shell. However, treatments with guar gum incorporating 50, 100, and 200 μL of PBEO effectively mitigated this darkening effect after 60 days of storage, as evidenced by their L* values.

The a* color index, representing the green‐red chromaticity (with positive and negative values indicating redness and greenness, respectively), exhibited a general decrease during storage (Table 6), suggesting a shift toward a greener hue. This observation aligns with the findings of (Hashemi et al. 2021a, 2021b) and Molamohammadi et al. (2020), who also reported a reduction in a* values in fresh pistachios during storage.

The Δ*E* in uncoated and coated fresh pistachio shells during 60 days of storage is presented in Table 6. While Δ*E* generally increased over time, pistachios coated with guar gum in combination with PBEO exhibited significantly greater total color variation after 60 days compared to other treatments.

### 3.9 The Effect of Guar Gum and PBEO at Different Times on the Number of Molds and Microorganisms in *Pistacia vera*

Our study revealed a significant increase in mold and yeast growth on pistachios in the control group, escalating from 10 colony‐forming units (CFU) on the first day of storage to 465 CFU at the end of storage. However, the application of guar gum at a concentration of 0.25% effectively suppressed this growth, maintaining mold and yeast counts at 10 CFU and consequently enhancing the storage quality of pistachios until Day 40 (Table 7). Furthermore, microbial growth, encompassing a broader spectrum of microorganisms, also exhibited a substantial increase in the control group, rising from 128 CFU on the first day of storage to 350,000 CFU at the end of storage. Notably, the application of PBEO at concentrations of both 50 μL and 200 μL demonstrated the most significant effect in inhibiting microbial proliferation, reducing counts to 10 CFU in both treatments. This effectively improved the storage stability of pistachios at 40 days of storage (Table 7).

**TABLE 7:** The effect of guar gum and PBEO at different times on mold and microorganism levels.

Time (days)	Guar gum (%)	Essential oil (μL)	Mold and yeast (CFU)	Microorganism (CFU)
15	Control	Control	10v	128o
		50	10v	128o
		100	10v	128o
		200	10v	128o
	0.25	Control	10v	128o
		50	10v	128o
		100	10v	128o
		200	10v	128o
	0.5	Control	10v	128o
		50	10v	128o
		100	10v	128o
		200	10v	128o
	1	Control	10v	128o
		50	10v	128o
		100	10v	128o
		200	10v	128o
40	Control	Control	950m	1,590,000c
		50	10v	10p
		100	10v	658m
		200	10v	10p
	0.25	Control	10v	20,870L
		50	704n	5874k
		100	498p	19,700L
		200	142t	500,000d
	0.5	Control	10v	6800k
		50	10v	315,000f
		100	10,125b	365,000f
		200	2520g	72,000i
	1	Control	10v	390,000f
		50	618o	39,950e
		100	2310h	516,000d
		200	118u	137,500h
60	Control	Control	1580j	5,225,000a
		50	10v	10p
		100	10v	318m
		200	10v	10p
	0.25	Control	10v	325000f
		50	8524c	1210l
		100	950l	190n
		200	3987e	54,000j
	0.5	Control	427r	468,000e
		50	1150k	410,000e
		100	2870f	500,000d
		200	4256d	43,600k
	1	Control	465q	350000f
		50	1940i	221,500g
		100	154s	365,000f
		200	17,500a	1,850,000b

*Note:* Different letters in the same column indicate significant differences (*p* < 0.05).

Abbreviation: CFU = Colony‐forming unit.

### 3.10 The Effect of Different Concentrations of PBEO on Different Days on the Growth of Aspergillus and Penicillin Fungi in *Pistacia vera*

Our study demonstrated a significant increase in *Penicillium* fungal growth on pistachios in the control group, with the diameter of fungal colonies expanding from 13.33 mm on the first day of storage to 25.66 mm at the end of storage. However, the application of PBEO at a concentration of 200 μL effectively suppressed this growth, reducing the colony diameter to 6.66 mm at the end of storage, thereby enhancing pistachio storage until 60 days (Table 8). Similarly, *Aspergillus* fungal growth on pistachios also increased in the control group, with colony diameters rising from 22.6 mm on the first day of storage to 25.6 mm at the end of storage. Nevertheless, the application of PBEO at a concentration of 50 μL proved most effective in inhibiting *Aspergillus* growth, reducing the colony diameter to 18.66 mm at the end of storage, which consequently improved the storage stability of pistachios until the sixtieth day (Table 8).

**TABLE 8:** The effect of different concentrations of PBEO on the growth of *Penicillium* and *Aspergillus* fungi over various days.

Day	Essential oil (μL)	Penicilium (diameters)	Aspergillus (diameters)
	Control	13.33bc	22.6gfedc
1st	50	12.33bc	17i
	100	11.66bcd	18.3ih
	200	13.66b	20 ihg
	Control	13.66b	24edcb
15th	50	12.33bc	23gfedc
	100	14.3b	25.6abcd
	200	10.33ebcd	26/6abc
	Control	13.66b	24.3fedcb
40th	50	14.3b	25abcd
	100	12.66bc	25.6abcd
	200	11bcd	28.66a
	Control	14.3b	24.6edcb
50th	50	12.66bc	20.3ihgf
	100	13bc	24.3fedcb
	200	13.66bc	24fedcb
	Control	25.66a	25.6dcba
60th	50	9.33cde	18.66ih
	100	8 def	21.6hgfed
	200	6.66ef	21hgfe

*Note:* Different letters in the same column indicate significant differences (*p* < 0.05).

### 3.11 The Effect of Different Concentrations of Guar Gum on the Solid Soluble Matter of Fresh *Pistacia vera*

**FIGURE 5:**
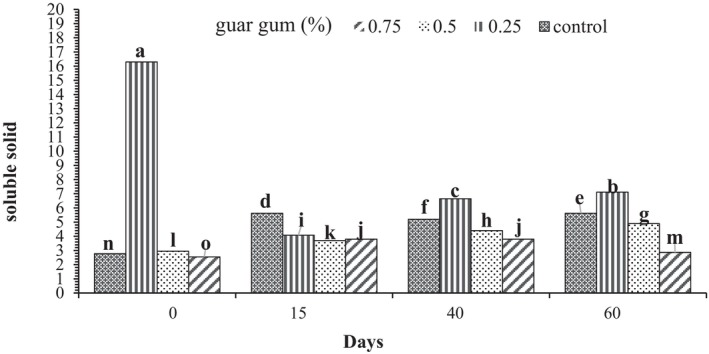
The effect of different concentrations of guar gum on the solid soluble matter of fresh *pistacia vera* in various days.

Our research indicated a transient increase in the number of soluble solids in pistachios within the control group during 60 days of storage. However, a significant decrease in soluble solids was subsequently observed within the control group between Days 15 and 60. Notably, the application of guar gum effectively mitigated the increase in soluble solids, resulting in a lower overall soluble solids content and, consequently, contributing to enhanced quality and improved storage of pistachios until Days 40–60 (Figure 5).

## 4 Discussion

EOs are natural compounds derived from plants that possess antioxidant, antimicrobial, and preservative properties and are widely used to improve quality and extend shelf life in the postharvest industry of vegetables and fruits. This study examines the effect of PBEO and *Cyamopsis tetragonoloba* L on the shelf‐life extension of pistachio fruits. In this study, the GC–MS device was used to identify the major compounds in PBEO, with E‐beta‐ocimene being the predominant component at 54.3%. Hosseaini et al. (2022) reported in a similar investigation that the PBEO primarily consists of the important compounds myristicin (21.1%), cis‐isomyristicin (17.2%), E‐β‐ocimene (11.1%), and Z‐β‐ocimene (6.2%) myristicin (21.1%), cis‐isomyristicin (17.2%), E‐β‐ocimene (11.1%), and Z‐β‐ocimene (6.2%). The EO profiles obtained in this study exhibited notable quantitative and qualitative discrepancies compared to previously published data, potentially stemming from variations in the plant's genetic background (species/ecotype) and environmental factors associated with its geographical location (Heydari‐Majd et al. 2019a, 2019b).

Plant oils exhibit diverse characteristics based on species and fatty acid composition, necessitating their production for specific applications. Similarly, fatty acid profiles dictate the potential uses of plant oils. GC‐FID analysis of guar gum revealed oleic acid (18:1) and palmitic acid (16:0) as the predominant fatty acids, with unsaturated fatty acids (73.58%) significantly exceeding saturated fatty acids (26.3%). These findings align with those of Akcura et al. (2019), who reported oleic acid levels ranging from 20.07% to 33.62% and linoleic acid ranged from 31.36% to 40.72% in guar gum. Although guar seeds contain relatively low lipid concentrations, their oil possesses qualitative attributes that suggest its potential as an edible oil source. The high content of essential fatty acids and favorable quality indices of guar seed oil are comparable to those of common edible oils like cottonseed, soybean, and corn oil, making its lipid fraction suitable for both animal and human nutrition. Notably, the sum of long‐chain saturated fatty acids (SFAs) such as arachidic, behenic, and lignoceric acids was quite low. This is nutritionally significant, as oils with high levels of long‐chain SFAs have been reported to be less digestible in both humans and animals.

FTIR spectroscopy confirmed the presence of key functional groups in guar gum, including O–H, C–H, C = O, and C–O–C. These groups are essential for hydrogen bonding, the complex galactomannan structure, and the rheological properties of the guar gum. These results are consistent with previous studies by Verma et al. (2025) and Poojashree et al. (2025), which also identified similar functional groups in guar gum.

The results of this study revealed that the incorporation of PBEO significantly (*p* < 0.05) affected the PS and ζ of the coating solutions. Smaller PS are known to enhance stability by minimizing sedimentation and aggregation, as uniformly dispersed particles are less prone to clumping, thereby maintaining a consistent coating throughout the application process. The ζ, which measures the surface charge of particles in suspension, provides insight into charge attraction/repulsion and electrostatic interactions between matrix components. A high absolute zeta potential value (either positive or negative) indicates strong electrostatic repulsion between particles, which can prevent aggregation and enhance stability, ultimately contributing to a uniform coating. This is crucial for achieving optimal coating performance, as it ensures that the coating material remains stable and evenly distributed during the application process. Prior studies have indicated that combining EOs with biopolymers can enhance matrix stability and colloidal dispersion through hydrophobic‐hydrophilic interactions (Kumar et al. 2024; Stoleru and Brebu 2021). Shinde et al. (2024) also investigated the influence of EOs on the PS and ζ potential of coating formulations. Their findings revealed that increasing concentrations of oregano EO led to an increase in the PS of guar gum‐almond gum coatings, attributed to hydrophobic‐hydrophilic interactions between the EO and the coating matrix. In general, the impact of incorporating plant EOs on the ζ potential and PS of guar gum solutions used for fruit coatings appears multifaceted and contingent on factors such as the type and concentration of EO, as well as the specific composition of the guar gum solution. Further investigation is warranted to elucidate these complex relationships.

The contact angle (CA) with water is a quantitative measure of the superficial hydrophilicity of coating matrices. A lower CA value indicates a higher density of accessible hydrophilic groups on the surface. However, the incorporation of PBEO led to a significant increase in CA, suggesting a reduction in surface hydrophilicity. The oily nature of plant EOs generally leads to increased CA, indicating enhanced hydrophobicity. This has been extensively demonstrated in research on edible films and coatings containing EOs. As an example, Heidare‐Majd et al. (2022) found that incorporating *Zataria multiflora* L. and *Mentha piperita* *L. EOs* into PLA films resulted in a higher CA (Heydari‐Majd et al. 2022).

This study demonstrated that the application of PBEO and guar gum significantly preserved the quality of pistachio kernels during storage. Lipid peroxidation, a primary driver of oxidative processes, is initiated by reactive oxygen species (Hu et al. 2025). Our findings indicate that PBEO‐based guar coatings effectively inhibited lipid peroxidation in pistachios during storage, likely due to the antioxidant compounds present in the EO. Natural EOs are known to exhibit peroxide‐scavenging activity (PV) due to their antioxidant constituents (Sabaghi et al. 2015; Habibi et al. 2023). The antioxidant mechanism of plant EOs involves metal ion chelation and hydrogen donation to oxygen radicals, protecting free fatty acids (FFA) from oxidative damage by neutralizing free radicals and reactive oxygen species (Hu et al. 2025). Previous studies have also shown that plant EOs prevent and inhibit lipid peroxidation in pistachios (Shakerardekani et al. 2021; Hashemi et al. 2021a, 2021b; Nejatian et al. 2023).

The levels of phenolic compounds and total antioxidant capacity in fresh fruits are influenced by factors such as analytical methods, cultivar, species, geographical origin, cultural practices, and environmental conditions. Phenolic compounds, synthesized via the phenylpropanoid pathway (Hu et al. 2025), decreased during storage in both control and PBEO‐guar gum‐coated pistachios. This decline is likely due to cell wall degradation, enzyme activity, and postharvest decay (Hashemi et al. , 2021a, 2021b). However, the PBEO‐guar gum treatment resulted in the preservation of phenolic compounds and antioxidant capacity compared to uncoated samples, consistent with previous reports on pistachios (Shakerardekani et al. 2021; Nejatian et al. 2023) and walnuts (Habibi et al. 2023). This preservation may be attributed to the production of secondary metabolites by the coating and its ability to limit oxygen exposure, thereby inhibiting enzymes responsible for phenolic compound degradation (Habibi et al. 2023). The observed preservation of total antioxidant activity in treated samples is likely linked to the protection of phenolic and antioxidant compounds by the EOs.

Acid values, indicative of free fatty acid content, were lower in treated samples compared to the control, suggesting reduced enzymatic hydrolysis of lipids. Furthermore, the PBEO treatment helped maintain total soluble solids in pistachios throughout the storage period. These findings align with previous studies demonstrating that plant EOs can inhibit acid value increases and preserve total soluble solids during storage (Chatrabnous et al. 2018; Zibaei‐Rad et al. 2024), contributing to improved product quality, stability, and sensory properties.

The PBEO treatment also influenced pistachio kernel firmness by modulating moisture retention, controlling enzymatic activity, and enhancing oxidative stability. Treated pistachio kernels exhibited greater firmness and reduced moisture loss compared to the control, likely due to the formation of a protective barrier that minimizes water loss, limits oxidative degradation of cell wall components, and preserves structural integrity. The antimicrobial properties of the EOs may also inhibit microbial enzymatic activity, preventing cell wall degradation and tissue softening (Hashemi et al. 2021a, 2021b; Nejatian et al. 2023).

The observed decrease in color indices (L* and a*) suggests an increase in browning reactions and the degradation of anthocyanin pigments present in the pistachio hull (Molamohammadi et al. 2020). Specifically, the reduction in a* values in treated samples can be attributed to anthocyanin degradation. Oxygen promotes browning in fruit pericarps by accelerating anthocyanin breakdown and the oxidation of phenolic compounds by polyphenol oxidase enzymes (Hashemi et al. 2021a, 2021b). Guar gum is hypothesized to act as a barrier against gas transfer, thereby reducing surface browning by limiting oxygen availability. After 60 days of storage, the untreated control exhibited the lowest L* value, indicating an undesirable darker color. All guar and EO treatment combinations resulted in higher L* values compared to the control, suggesting a protective effect on shell lightness. Similarly, chitosan coatings have been shown to reduce color changes associated with fruit ripening, possibly by reducing O_2_ and increasing CO_2_ levels (Molamohammadi et al. 2020). Hashemi et al. 2021a, 2021b demonstrated the effectiveness of gum Arabic as an edible coating in preserving L* and a* values, attributing its effect to a reduction in respiration rate and delayed ripening in pistachio hulls.

The plant extract and EOs reduced yeast and mold populations in pistachios (Shakerardekani et al. 2021; Hashemi et al. 2021a, 2021b; Nejatian et al. 2023) and walnut kernels (Habibie et al. 2019). The antimicrobial activity of EOs is attributed to their acidic nature and structural composition, leading to proton release, intracellular potassium leakage, and cytoplasmic membrane damage under acidic conditions. Our results demonstrate that the PBEO effectively inhibited microbial activity in pistachios. The low molecular weight and hydrophobic nature of EOs facilitate their penetration of the cell membrane, disrupting its function (Nazzaro et al. 2017). Terpenoids and terpenes present in EOs also contribute to mitochondrial dysfunction, ATP level alterations, and increased reactive oxygen species (Haque et al. 2016). Edible coatings also can function as gas barriers, thereby altering the internal atmosphere surrounding the fruit. This passive modification of the atmosphere can inhibit microbial growth, particularly fungal decay, in fresh pistachios (Molamohammadi et al. 2020). Edible coatings can act as a gas barrier, thus modifying the internal atmosphere in the fruit. The creation of a passive‐modified atmosphere around the fruit can inhibit the growth of microorganisms, especially fungal decay in fresh pistachios. In conclusion, PBEO‐loaded guar gum effectively reduced oxidative and enzymatic degradation by lowering acid value, maintaining phenolic compounds and soluble solids, enhancing antioxidant activity, and forming a protective barrier. The antimicrobial properties of PBEO further inhibited microbial enzymatic activity, preserving pistachio firmness. This research confirms that the PBEO‐loaded guar coating extended pistachio shelf life from 8 to 16 days based on microbial counts. Future studies should investigate the effects of varying EOs concentrations on aroma, flavor, and sensory acceptance. Exploring the precise molecular mechanisms of EOs action on cellular structure and assessing their impact under industrial‐scale conditions would further optimize their application in pistachio processing.

## Author Contributions

**Mohammed Hamdan Aldarraji:** conceptualization (lead), methodology (supporting). **Hatem Mohammed Hasan:** data curation (lead), methodology (supporting). **Hossein Talepour Ardekani:** investigation (supporting), formal analysis (supporting). **Mojtaba Heydari‐Majd:** original draft (supporting), methodology (supporting). **Heidar Meftahizade:** data curation (lead), original draft (lead), project administration (lead) data curation (supporting), review and editing (supporting). **Mansour Ghorbanpour:** investigation (supporting), review and editing (lead).

## Ethics Statement

The authors have nothing to report.

## Conflicts of Interest

The authors declare no conflicts of interest.

## Data Availability

The data and materials will be available on request.
